# Renewable Lignosulfonate-Assisted Synthesis of Hierarchical Nanoflake-Array-Flower ZnO Nanomaterials in Mixed Solvents and Their Photocatalytic Performance

**DOI:** 10.1186/s11671-016-1474-x

**Published:** 2016-05-21

**Authors:** Yue Li, Hong-Fen Zuo, Yuan-Ru Guo, Ting-Ting Miao, Qing-Jiang Pan

**Affiliations:** Key Laboratory of Bio-based Material Science and Technology (Ministry of Education), College of Material Science and Engineering, Northeast Forestry University, Harbin, 150040 China; Key Laboratory of Functional Inorganic Material Chemistry (Ministry of Education), School of Chemistry and Materials Science, Heilongjiang University, Harbin, 150080 China

**Keywords:** ZnO nanomaterials, Mixed solvents, Sodium lignosulfonate, Catalytic properties, Hierarchical structure

## Abstract

With the assistance of sodium lignosulfonate, hierarchical nanoflake-array-flower nanostructure of ZnO has been fabricated by a facile precipitation method in mixed solvents. The sodium lignosulfonate amount used in our synthetic route is able to fine-tune ZnO morphology and an abundance of pores have been observed in the nanoflake-array-flower ZnO, which result in specific surface area reaching as high as 82.9 m^2^ · g^−1^. The synthesized ZnO exhibits superior photocatalytic activity even under low-power UV illumination (6 W). It is conjectured that both nanoflake-array structure and plenty of pores embedded in ZnO flakes may provide scaffold microenvironments to enhance photocatalytic activity. Additionally, this catalyst can be used repeatedly without a significant loss in photocatalytic activity. The low-cost, simple synthetic approach as well as high photocatalytic and recycling efficiency of our ZnO nanomaterials allows for application to treat wastewater containing organic pollutants in an effective way.

## Background

With strengthening consciousness of environmental protection and effective utilization of resources, much more attention has been paid to using renewable materials to synthesize highly efficient catalyst for sewage treatment [1]. As a low-cost renewable biomass resource, lignin is the most abundant organic material in nature [2]. It is derived from black liquor generated in the pulping process. Every year, tens of million tons of lignin are produced all over the world [3] Unfortunately, most of them are discarded as waste, greatly contaminating our environment, while only a small part is inferiorly used. Therefore, exploration on high-effective utilization of lignin not only makes good use of natural resource but also protects the environment from pollution of black liquor [4, 5].

Sodium lignosulfonate (SL for short) is one of the most important derivatives of lignin. Lignosulfonate can be obtained by the sulfonated modification of lignin. Moreover, SL itself is a by-product of sulfite pulp and paper industry. It is a biomacromolecule polyelectrolyte and can act as an anionic surfactant [6], because of its highly cross-linked polymers formed by various hydrophobic phenylpropanoid units and diverse hydrophilic groups. Various active functional groups ensure its favorable water solubility and effective surface charges. In different solvents, SL can form associations and the degree of associations changes according to the environment of the solution (such as SL concentration, the type of the solvents, and ion strength). The aggregation state of SL will change as a result of the existence of electrostatic repulsion [7–9]. Spheroidal shape and irregularly disk-like shape of SL in different conditions were corroborated by previous works [10]. The layer-by-layer self-assembly of SL can also easily be produced in saline solution because of its capability of binding transition metal ions such as Cu^2+^ and Zn^2+^. [11]. All of these outstanding performances have enabled SL to be applied in the synthesis of nanoparticles of transition metal oxide.

ZnO, as a typical transition metal oxide, has recently attracted increasing attention in view of its outstanding properties [12–15]. All these provide ZnO with diverse applications in dye-sensitized solar cell, nanoimprint lithography, drug delivery, and photocatalysis. Particularly, ZnO has excited much interest of researchers because of photocatalytically degrading organic pollutants and is becoming more promising alternative to TiO_2_.

It has well established that properties and performance of ZnO strongly depend on its structure and morphology [16]. A variety of ZnOs have been fabricated to meet practical applications [17–19]. More fascinating merits have been found for hierarchical three-dimensionally (3D) porous micro-/nano-architecture ZnO, which is self-assembled by low-dimensional nano-sized building blocks. The high porosity of hierarchical 3D ZnO greatly facilitates gas diffusion and mass transport in sensor and surface chemical reaction materials [20]. However, most of the synthetic methods for these complex structures are restricted because of requiring rigorous conditions.

Apart from morphology discussed above, specific surface area is also one of the important factors to affect ZnO properties. So far, the specific surface area of commercial ZnO is very low, ranging 4–5 m^2^ · g^−1^. Using highly ordered mesoporous carbon templates, Polarz and co-workers synthesized ZnO of 195 m^2^ · g^−1^ [21]. Larger surface area of 305 m^2^ · g^−1^ was achieved by Goswami et al., in which the use for drug delivery was emphasized and synthetic details were not clearly described [22]. Although with relatively high surface area, the above ZnO does not show the hierarchical architecture. Currently, lower than 50 m^2^ · g^−1^ specific surface area has been reported for ZnO having the hierarchical structure [23–25]. Therefore, it is highly demanded to synthesize ZnO materials having both 3D hierarchical architecture and high specific surface area, especially in the case of using a simple and economical approach with a low-cost surfactant.

In our previous work, we have developed a preparation method of ZnO using the SL [26, 27]. The prepared ZnO shows good catalytic performance with specific surface area about 20–30 m^2^ · g^−1^. On the basis of previous work, the mixed solvent approach xhas been developed for the synthesis of ZnO nanomaterials. Hierarchically porous flake-array-flower structure with high specific surface area has been successfully fabricated. Its photocatalytic performance was tested, and the corresponding growth process and possible mechanism have been proposed. Herein, we have used the cost-effective SL surfactant, derived from industrially discarded lignin. So, this study allows for not only effective utilization of resources and protection of environment but also a good way of massively producing hierarchical porous nanomaterials of ZnO.

## Methods

In this work, ZnO architectures were synthesized by a surfactant-assistant solution-based method. Zinc acetate (Zn(OAc)_2_ · 2H_2_O) (Fuchen chemical factory, Tianjin) was taken as Zn^2+^ source and sodium hydroxide (NaOH) (Tianda Chemical Corp., Tianjin) was used as the precipitating agent. SL (Tumen Qianjinfuli chemical Co. Ltd.) was used as surfactant material. According to manufacturer’s specifications, the parameters of the SL is that pH = 4.5–6, water content ≤8.5 %, insoluble matter ≤1.0 %, sugar content ≤12.0 %, Ca and Mg content ≤1.5 %, and inorganic salt (Na_2_SO_4_) ≤5.0 %.

In a typical experiment, 7 g of SL was added to 50 mL of deionized water to form the surfactant solution. And 3.5 g of Zinc acetate (0.53 M) was dissolved to 30 mL deionized water forming the Zn^2+^ solution. These two solutions were mixed under stirring and then 100-mL anhydrous ethanol was added to this mixed solution. We added 20 mL of NaOH (2.5 M) solution into the above mixture dropwise. The final solution was kept stirring for 30 min to form a homogeneous precursor solution. Finally, the precursor solution was kept stirring in 80 °C water bath for 5 h. Precipitate was harvested by centrifugation and washed and dried at 50 °C. Then, the as-obtained powders were calcined in air atmosphere at 500 °C for 2 h to obtain the final ZnO product.

X-ray diffraction (XRD) patterns were recorded on a Rigaku D/MAX-RC diffractometer using Cu Kα radiation, and scans were performed from (2θ) 5° to 80° by rate 4°/min. Scanning electron microscopy (SEM) images were taken with a field emission microscope FEI Sirion. The transmission electron microscopy (TEM) images and high-resolution TEM (HRTEM) images of the samples were performed on a JEM-2100 electron microscope (JEOL, Japan) with an acceleration voltage of 200 kV. Carbon-coated copper grids were used as the sample holders. Brunauer–Emmett–Teller (BET) nitrogen adsorption–desorption experiments were carried out on the automated surface area and pore-size analyzer (ST-2000).

The photocatalytic activity of the prepared hierarchical ZnO to decompose the methylene blue (MB) was investigated. In the experiment, 0.1 g ZnO was dispersed in 40 mL of MB (10 mg · L^−1^) solution. And the stirring suspensions were exposed to the UV irradiation (6 W, 365 nm, WFH-203) under ambient conditions. After being kept in the dark for 30 min, the solution reaches the absorption-desorption equilibrium. The distance between UV light and the photoreaction vessel is 12 cm. In order to evaluate the efficiency of the degradation processes, the suspension was analyzed at a definite time interval, by recording variations at the maximum absorption around λ = 664 nm using a UV–vis spectrophotometer (Shanghaijingmi instrument Co., Ltd. UV762).

## Results and Discussion

### XRD Measurement

**Fig. 1:**
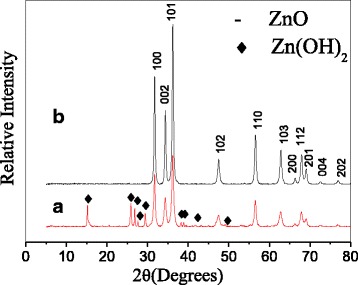
XRD patterns of ZnO (**a**) non-calcined (*red line*) (**b**) calcined (*black line*) at 500 °C for 2 h

X-ray diffraction (XRD) patterns were recorded to investigate the phase and purity of the synthesized product. We have illustrated in Fig. 1 the XRD patterns of the synthesized ZnO with and without calcination. As seen in Fig. 1a in the case of non-calcination, except for characteristic peaks of ZnO phase, the diffraction peaks of Zn(OH)_2_ are also present (JCPDS No. 20-1435). Previous reports indicated that the direct synthesis of zinc oxide would result in impurity of more or less zinc hydroxide at a low temperature [12]. Subsequent calcination enables the XRD pattern to exhibit pure ZnO characteristic peaks. It is illustrated from Fig. 1b that all the diffraction peaks can be well indexed as the hexagonal wurtzite crystalline structure (JCPDS No. 36-1451). This also confirms that precursor of zinc hydroxide phase has been fully decomposed to ZnO. Meanwhile, one can see that, upon calcining, peak intensities of ZnO are obviously enhanced compared to those of non-calcined ZnO. This testifies that pure and well crystallinity of ZnO would be obtained by calcination. The mean crystallite size of calcined ZnO was computed to be about 21.8 nm via measurement of the full width at half-maximum (fwhm) of ZnO (101) diffraction peak using Scherrer formula.

### Synthetic Condition and SEM

#### Effect of SL on ZnO Morphology

**Fig. 2:**
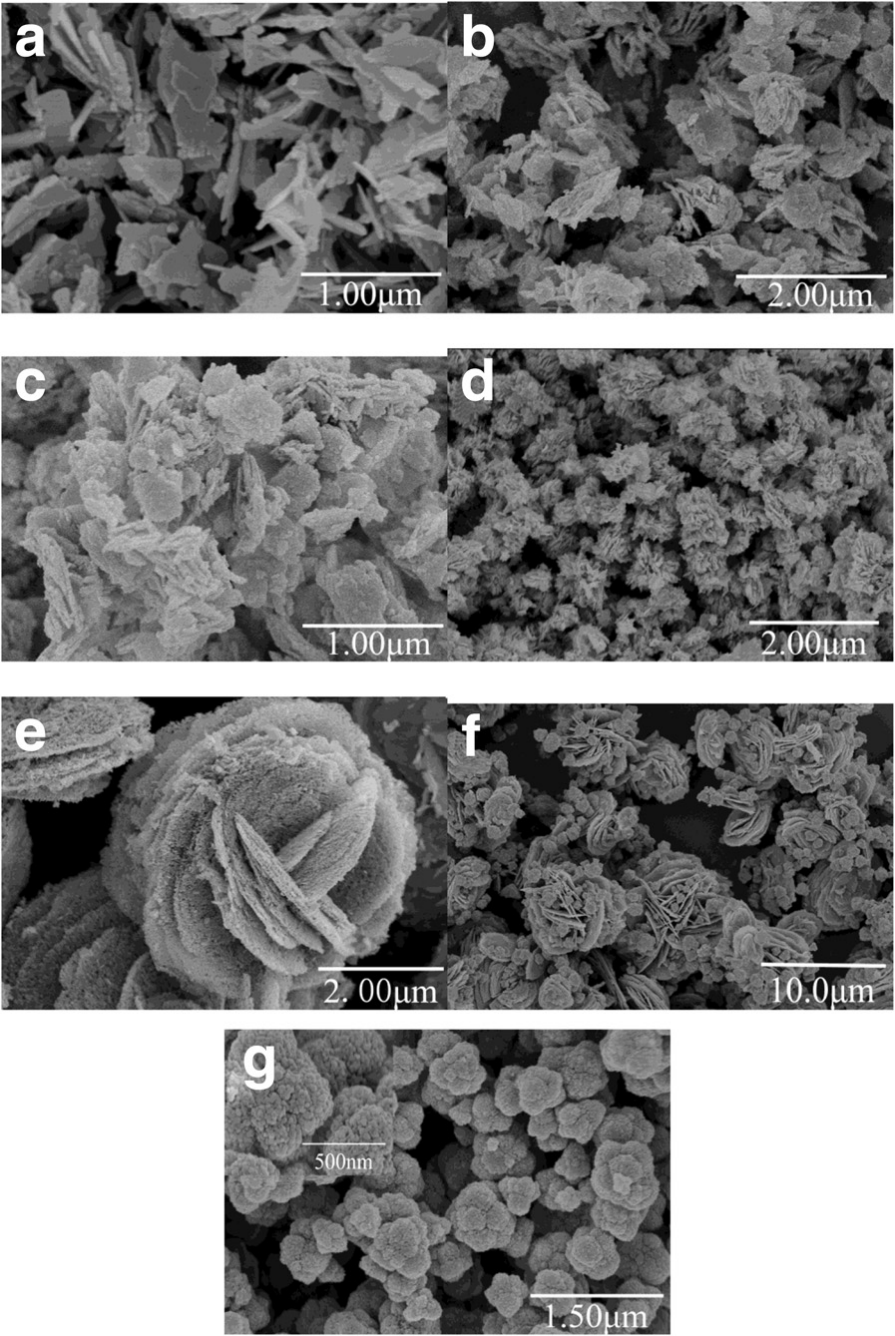
SEM images of ZnO with amount of SL of: (**a**) 0 g, (**b**) 1 g, (**c**) 3 g, (**d**) 5 g, (**e**) 7 g (**f**) 9 g, and (**g**) 11 g

It is well known that the added amount of surfactant has a significant effect on morphology of formed nanomaterials. To obtain a suitable architecture of ZnO that is helpful for subsequent photocatalysis, a series of experiments were performed with the variation of amount of SL from 0 to 11 g. Building on above XRD results, all these prepared samples will be calcined to obtain well-crystallized ZnO. Their SEM images are presented in Fig. 2. When we do not add the SL surfactant in the synthesis, farfetched ZnO fragments were obtained (Fig. 2a). Upon adding 1–3 g SL, relatively small slices of ZnO were formed (Fig. 2b, c), albeit with irregular shape. These desultory slices were transformed into flocculent ZnO clusters when 5 g SL was used during the synthesis (Fig. 2d). In the case of increasing amount of SL to 7 g, we obtained 3D hierarchical ZnO architectures in Fig. 2e. This sample is featured with flower-like morphology. Moreover, some pores were observed on it. With further increasing SL to 9 g (Fig. 2f), many ZnO particles were emerged although still having partial micro-flower structure. It was observed that morphology like broccoli in nature is formed when more SL was added (11 g, in Fig. 2g). It shows a relatively broad size distribution of 300~750 nm, resulting from agglomeration of sub-crystals. Our present studies indicate that the added amount of SL can fine-tune the morphology of synthesized ZnO. The 7 g SL is optimal amount to prepare the uniform hierarchical flower-like ZnO architectures.

#### Calcination and Cosurfactant of Ethanol

**Fig. 3:**
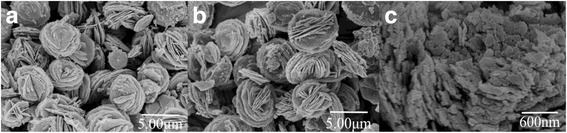
SEM images of the 7 g SL synthesized ZnO with conditions of (**a**) calcination and ethanol, (**b**) *non*-calcination and ethanol, and (**c**) calcination and *no* addition of ethanol

In the assistance of 7 g SL surfactant, we will discuss effect of the calcination and the use of cosurfactant of ethanol on morphology of synthesized ZnO nanomaterials. First, the calcining procedure is addressed while the ethanol cosurfactant is used in the synthesis. Morphologies of calcined and non-calcined ZnO have been pictured in Fig. 3a, b, respectively. These SEM images indicate that both products consist of a large number of three-dimensional (3D) flower-like microsized ZnO with the mean size of 4–5 μm. Meanwhile, all ZnO flowers show plentiful pores on every petal. Comparatively, smooth surface is observed for a part of assembled microflowers in the non-calcined ZnO except porous flower ZnO (Fig. 3b). The smooth surface may be composed by Zn(OH)_2_, SL, and ZnO composites. And it will produce more porous ZnO on the flower petal after calcination.

The effect of ethanol on the formation of the ZnO hierarchical flower-like structure has been examined as seen in Fig. 3a, c. Also, both optimal 7 g SL surfactant and calcination were used. Addition of ethanol to the reaction solution results in much more regular ZnO slices in Fig. 3a than those in Fig. 3c. It is rationalized that ethanol raised surface activity of SL and enhanced its coordination to Zn^2+^ ions. At the same time, once nuclei of ZnO were formed in the solution, the presence of ethanol decreases solubility of ZnO, which makes it easy to form small size nanostructure and eventually produces a hierarchical structure.

### Structural Properties of Optimal Sample

**Fig. 4:**
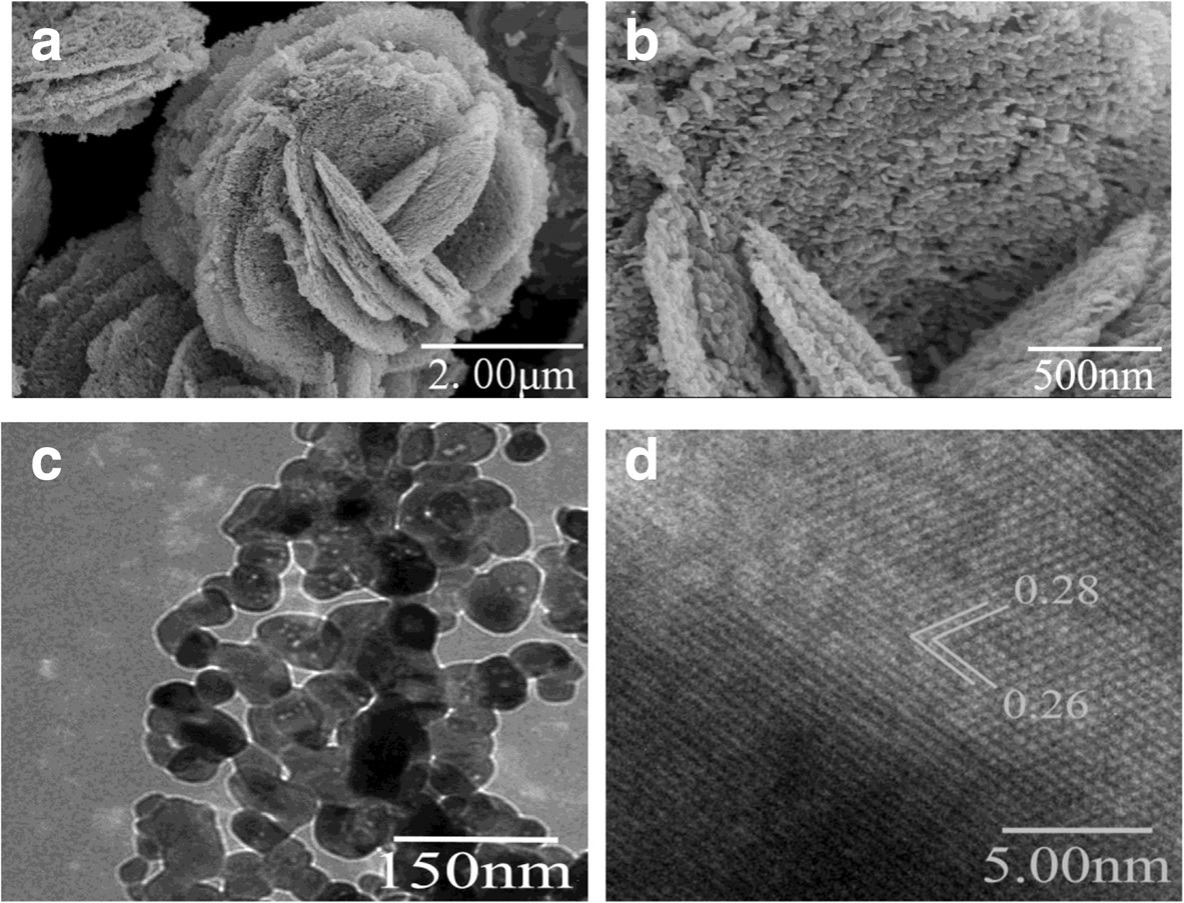
Images of the hierarchical flower-like ZnO nanostructure: **a** high-magnification SEM, **b** high-magnification SEM with 500 nm, **c** TEM, and **d** HRTEM

And thus, the sample was synthesized with the 7 g SL and ethanol cosurfactant, followed by a calcining procedure. Closer inspection (Fig. 4a, b) reveals that the surface morphology of single cambered lamina is composed of numerous ZnO nanoflake array. These nanoflake arrays almost stand vertically on the 2D lamina and cross-link and even overlap with each other by the edge to form networks as porous lamina structures. This special structure was also caused by the hydrolysis of the initial Zn(OH)_2_ matrix as well as the shrink of ZnO crystals during the calcination. It is consistent with XRD results and previous works about the hierarchical porous ZnO structures by calcining [Zn_5_(CO_3_)_2_(OH)_6_] precursor. Herein, we developed a method with introducing derivative of industrially discarded waste to synthesize hierarchical porous ZnO architectures.

Further detailed structural analysis of individual porous lamina was carried out using transmission electron microscopy (TEM) and high-resolution TEM (HRTEM). The TEM image of the edge part of single flower-like ZnO shows that each lamina is constructed from agglomerative nanoflakes, which is featured with a wide size distribution ranging from 20 to 80 nm (Fig. 4c). It is found that sizes measured from TEM are larger than those calculated by Scherrer’s equation according to XRD. This discrepancy is attributed to the aspect ratio for non-spherically shaped crystallites, as well as the FWHM by the microstrain in the crystallite [28]. These cross-linked nanoflakes show abundant porous structure, in accordance with the SEM results. Meanwhile, some mesopores with several nanometers in size can be clearly observed, which are scattering in the nanoflake array. Well-resolved two-dimensional lattice fringes observed in the HRTEM image (Fig. 4d) suggest good crystallinity of our synthesized nanoparticles. The interplanar distances between adjacent lattice planes were calculated to be 0.26 and 0.28 nm. They are attributed to *d*-spacing (002) and (100) planes of ZnO in the wurtzite phase, respectively.

**Fig. 5:**
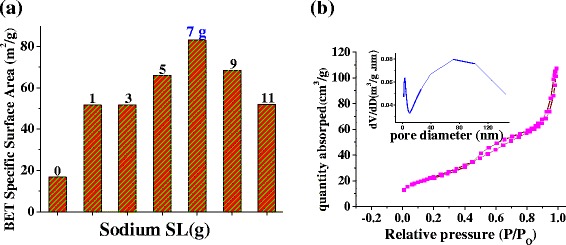
**a** Specific surface areas of ZnO synthesized by SL surfactant, whose amount is presented. **b** Nitrogen adsorption–desorption isotherms and the corresponding plots of Barrett–Joyner–Halenda (BJH) pore-size distribution for the porous ZnO synthesized with 7 g SL

Specific surface areas of the calcined ZnO prepared with different amount of SL were determined by BET method as shown in Fig. 5a. One can see that the BET-specific surface areas of ZnO are strongly affected by the added amount of SL used in the preparation. If SL is not added, then obtained area is as low as 16.7 m^2^ · g^−1^. In contrast, upon adding SL, even only 1 g, the specific surface area of synthesized ZnO will exceed 50 m^2^ · g^−1^. This results from SL guiding the formation of ZnO architecture. In assistance of 7 g SL, the synthesized nanoflake-array-flower ZnO has the largest specific surface area, reaching as high as 82.9 m^2^ · g^−1^. To our best of knowledge, the specific surface area of all the reported ZnO having the hierarchical structure remains lower than 50 m^2^ · g^−1^ [23, 25]. Notably, our ZnO displays much larger specific surface area, i.e., 30 m^2^ · g^−1^ larger than previously reported value.

Abundant pores in our synthesized ZnO materials are also evidenced by nitrogen adsorption-desorption isotherms and Barrett–Joyner–Halenda (BJH) pore-size distribution (Fig. 5b). The measurements find two kinds of pores in the nanoflake-array-flower ZnO. First is small mesopores, whose diameters range from 3 to 4 nm. They are formed by interconnected individual ZnO nanocrystallites. Meso and macropores are the second kind. Their diameters are relatively large, about 80 nm. These pores are constructed by nanoflakes’ interlacing, as observed in SEM images Fig. 4a, b).

### Formation Mechanism

It has been well established that SL is polyphenolic material, made from the copolymerization of three phenylpropanoid monomers such as coniferyl, sinapyl, and p-coumaryl alcohol. Due to containing both rich hydrophilic and hydrophobic sites, the macromolecular SL would form a structure of layer-by-layer self-assembly [11] in controlled experimental condition.

**Scheme 1:**
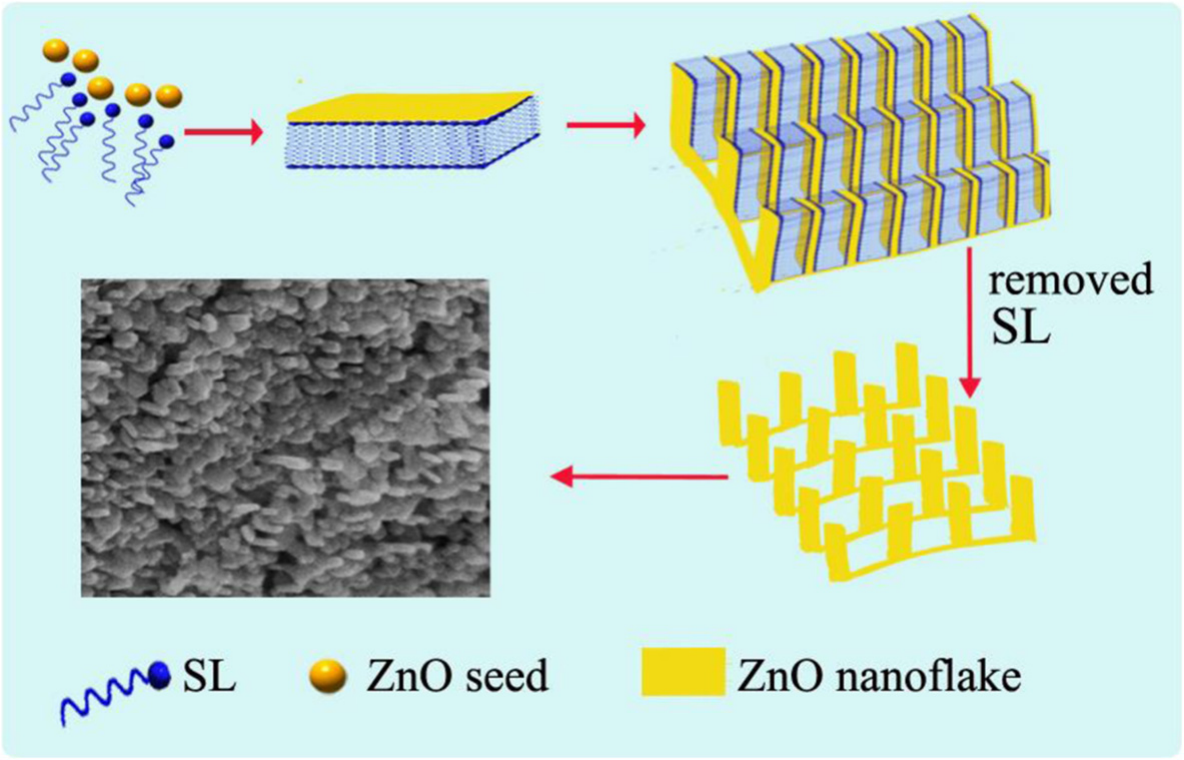
Formation of nanoflake-array-flower ZnO

As a polar crystal, ZnO is known to possess partially positively charged Zn^2+^-terminated (001) and negatively charged O^2−^-terminated (00$\overline{1}$) polar surfaces. It is evident that ZnO was reported to show the flower-like morphology [25, 29]. In the current experiment, it is deduced that the polar ZnO nanoseeds could be adsorbed onto the negative-charged surface of SL and then form the ZnO-SL composite with layer-by-layer structure. This shaped structure would induce nanoflakes to grow on the petal ZnO flower in a perpendicular direction. Meanwhile, electrostatic attractive interaction between the ZnO and SL layer are enhanced by ethanol co-solvent. Consequently, the flake-array hierarchical flower-like ZnO is constructed upon removing the SL. See the description of the process in Scheme 1.

The association degree and the aggregation shape of SL are significantly affected by the SL concentration [9]. For example, low concentration SL, using 1 ~ 3 g amount, shows relatively weak surface activity [13]; as a result, irregular slices of ZnO are formed [18]. Too much amount of SL (for instance 11 g) added into the reaction system would destroy the layer-by-layer structure of SL, yielding the broccoli-like ZnO [30]. In contrast, suitable SL concentration (7 g SL in 200 mL solvents) would retain surface activity as well as facilitate its lay-by-lay self-assembly. And hierarchical nanoflake-array-flower ZnO materials with high surface area are fabricated.

In brief, the formation of final ZnO architecture is attributed to a synergistic effect, i.e., the SL aggregation shape and the electrostatic attraction between SL and polar ZnO crystals. The absorption of ZnO enhances the layer-by-layer shape of SL, which in turn induces the ZnO to form flake-based structural unit and then self-assembly superstructures.

### Photocatalytic Performance

**Fig. 6:**
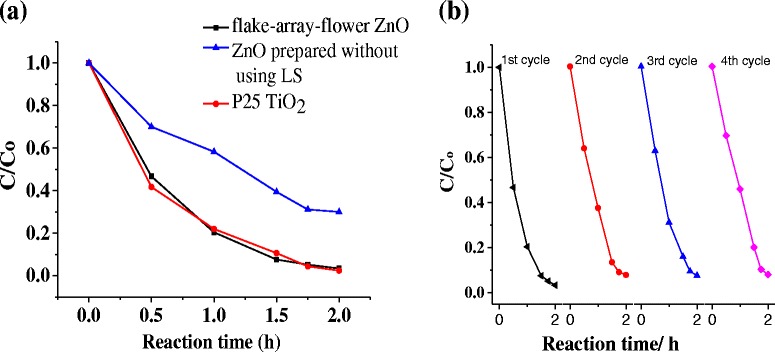
**a** Photocatalytic degradation curves of MB solution under UV illumination. **b** Re-using activity of ZnO to photodegrade MB

Given their porosity significantly facilitating mass transportation in photocatalytic process, hierarchically porous ZnO structures, especially with high specific surface area, are attractive to photocatalytically degrade organic pollutants in aqueous solution. The present hierarchical nanoflake-array-flower architecture is expected to exhibit excellent photocatalytic property. In the work, photocatalytic performance of the nanoflake-array-flower ZnO has been evaluated by photodegrading methylene blue (MB), which is a typical pollutant in the textile industry. By irradiation with UV lamination in low power (6 W), the degradation percentage of MB achieved 93 % within 1.5 h (Fig. 6a). Nearly all of the MB molecules were decomposed after 2 h. To further assess the photocatalytic activity, we have compared degradation ability of our ZnO with Degussa P25 TiO_2_ that is highly efficient photocatalyst. It is clearly seen from Fig. 6a that the nanoflake-array-flower ZnO shows a photocatalytic activity comparable to that of Degussa P25 TiO_2_, and even better in some cases. The present study indicates that our ZnO is actually superior to P25, as our ZnO was prepared with the renewable and cost-effective biomass SL surfactant. The utilization of this surfactant greatly protects the environment, for lignin is present in black liquor of industrial waste. Furthermore, the recycling photocatalytic performance of ZnO was also evaluated. In each circulation, the irradiating time is 2 h. It is shown in Fig. 6b that highly photocatalytic activity is well retained even after re-using four cycles. Therefore, our prepared flower-like ZnO may be highly promising for photocatalytic application due to its excellent photocatalytic activity, good re-using performance as well as low cost.

## Conclusions

A SL-assisted self-assembly of certain morphology of ZnO architecture has been achieved via a simple precipitation synthetic approach. Various morphologies of ZnO have been fabricated by fine-tuning amount of SL. It is revealed that a suitable amount plays an important role in self-assembly forming the hierarchical nanoflake-array-flower morphology of ZnO. The interesting architecture is featured with assembled lamina, while each lamina is constructed from agglomerative sub-level nanoflake array. A wide size distribution ranging from 20 to 80 nm was measured for these nanoflakes. It is observed in the flower-like ZnO that nanoflakes are standing perpendicularly to face of the lamina. All these structural arrangement has eventually resulted in a great increase of specific surface area of ZnO, reaching as high as 82.9 m^2^ · g^−1^. This surface area is much higher than those of reported ZnO materials with 3D hierarchical nanostructure. The formation mechanism of this nanostructure has been proposed as a synergistic effect of the SL aggregation shape and polar ZnO crystals.

The hierarchical nanoflake-array-flower ZnO has been examined to show a superior photocatalytic performance of degrading methylene blue, even under a very low-power UV illumination. This is attributed to both featured hierarchical nanostructure and high surface area of our ZnO. Its abundant pores and nanoflake array, simultaneously, may serve as scaffold microenvironments, which would enhance photocatalytic activity. It is found that photocatalytic efficiency of our ZnO is comparable to that of Degussa P25 TiO_2_. Moreover, a perfect durability in the photodegradation of MB has been observed for our hierarchical ZnO.

In the synthesis of hierarchical nanostructure ZnO, the cost-effective SL surfactant has successfully applied, allowing for a large-scale production. Moreover, SL is derived from renewable biomass lignin that is main component of black liquor from the pulping industry and is usually discarded as waste. So the use of SL would not only recycle industrial waste and protect environment but also extend application of renewable lignin in adding its value. In brief, our facile and low-cost synthetic approach is expected to be promising for the preparation of ZnO-based photocatalyst materials.
